# Efficacy and safety of Shatavari root extract in women with Polycystic Ovarian Syndrome: a randomized, double-blind, placebo-controlled trial

**DOI:** 10.3389/fendo.2026.1769773

**Published:** 2026-02-24

**Authors:** Yashodhara Mhatre, Pradeep Jadhav, Adwait Malik, Mayakalyani Srivathsan, Deepak Langade

**Affiliations:** 1 Department of Gynecology and Obstetric, ZOI Fertility, Mumbai, Maharashtra, India; 2 MGM Medical College and Hospital, Nerul, MGMIHS, Navi Mumbai, Maharashtra, India; 3 D. Y. Patil University of School and Medicine, Navi Mumbai, Maharashtra, India

**Keywords:** *Asparagus racemosus*, ovarian follicle, perceived stress scale, polycystic ovarian syndrome, Shatavari

## Abstract

**Background:**

Polycystic Ovarian Syndrome (PCOS) is a hormonal disorder affecting women of reproductive age. It frequently causes hormonal imbalance, irregular menstrual cycle, and in some cases, infertility. For centuries, traditional herbs like Shatavari (Asparagus *racemosus* Willd.) have been used to support women’s reproductive health, and some studies suggest it may help with PCOS symptoms. Thus, this study aimed to evaluate the safety and efficacy of standardized Shatavari root extract in women with PCOS.

**Methods:**

This prospective, randomized, double-blind, placebo-controlled trial was conducted on women aged 20–40 years for 12 weeks. Seventy participants were randomized into Shatavari root extract (SHT, n=35) and placebo (PL, n=35) groups. Sixty-six participants completed the full 12-week trial. The primary outcome was the change in ovarian and endometrial outcomes. Secondary outcomes were the change in Body Mass Index (BMI) and Perceived Stress Scale (PSS-10) scores. Assessments were performed at baseline, week 4, week 8, and week 12. Blood samples were collected at baseline and week 12 to estimate glycated hemoglobin (HbA1c), lipid profile, and serum insulin. The serum hormones, liver, renal, and thyroid functions were also assessed. For the safety assessment, adverse events were continuously monitored.

**Results:**

Baseline demographics and clinical parameters were comparable between groups. At 12 weeks, Ovarian volume did not differ significantly between groups (p= 0.254). SHT significantly reduced psychological stress (PSS score: -6.64 ± 3.99; p < 0.0001), decreased follicular count (p < 0.0001), and increased endometrial thickness (p = 0.028) compared to PL. No significant differences were observed in BMI, hormonal levels, or laboratory parameters. No serious adverse events occurred; mild to moderate events were reported in 11.4% (SHT) and 8.5% (PL) of participants, all manageable with standard therapy and not related to the intervention.

**Conclusion:**

Shatavari root extract oral administration can be a safe and effective potential intervention for women with PCOS. The study was registered with the Clinical Trials Registry of India (CTRI) under registration number CTRI/2024/10/074660 on October 3, 2024.

**Clinical Trial Registration:**

https://ctri.nic.in/Clinicaltrials/pmaindet2.php?EncHid=MTE0ODIy&Enc=&userName=, identifier CTRI/2024/10/074660.

## Background

Polycystic Ovarian Syndrome (PCOS) is a common endocrine disorder with a prevalence of 6–10% among women of reproductive age (12–45 years) (1). It is an anovulatory state, defined by irregular menstruation, disrupted ovulation patterns, and hormonal imbalance. These changes have an impact on fertility and overall reproductive health (2).

The current pharmacological options used for PCOS management include combined oral contraceptives (COCs), metformin, aromatase inhibitors (letrozole), and thiazolidinediones (pioglitazone, rosiglitazone). COCs are the first-line therapy of treatment for PCOS in women who are not planning for pregnancy, since they regularize menstrual cycles, reduce androgen levels, and improve acne and hirsutism. Since COCs may develop a risk of venous thromboembolism and can cause side effects like nausea, breast tenderness, and mood changes. Whereas metformin can improve insulin sensitivity and regularize the menstrual cycle, the evidence of its efficacy in non-obese women with PCOS is not clear. Aromatase inhibitors and thiazolidinediones have demonstrated promising results in ovarian induction, but their long-term use has raised concerns regarding their safety (3).

Considering these limitations, there is growing interest in complementary and alternative treatments, especially in plant-based remedies. Many such herbs contain bioactive compounds such as phytoestrogens (flavonoids, coumestans, lignans, and stibenes) and polyphenols, which are known to have biological activity on the female reproductive system (4).

One such herb is Shatavari (*Asparagus racemosus* Willd.), commonly known as “*Wild Asparagus*” belonging to the Asparagaceae family and revered in Ayurveda as the “Queen of herbs,” has gained scientific interest. It is native to the Indian subcontinent. It is classified as a ‘Rasayana’ in the Ayurvedic system of medicine and is known from past centuries for its benefits in improving reproductive health, enhancing physical and emotional resilience (5). The Shatavari roots are a rich source of plant steroidal saponins and estrogen modulators that may help in hormonal imbalances associated with PCOS. It is hypothesized that Shatavari supports the hypothalamic-pituitary-ovarian (HPO) axis and the functional integrity of the ovarian plexus by stabilizing hormone profiles and promoting menstrual regularity, which may help improve reproductive dysfunction (6).

The existing preclinical studies support this rationale. Vishnuvardhan et al. (2022) evaluated the alleviative effect of an aqueous extract of *Asparagus racemous* in a Wistar rat model of PCOS. The study assessed various hematological parameters, such as glucose, total protein, albumin, alanine transaminase (ALT), aspartate transaminase (AST), alkaline phosphatase (ALP), triglycerides, cholesterol, C-reactive proteins, BUN, and creatinine. Treatment with *Asparagus racemous* partially normalized these parameters, indicating a mitigating effect on PCOS-associated metabolic disturbances (7). Another study by Ghosh et al. (2025) investigated the therapeutic potential of *Asparagus racemosus* and *Vitex negundo* in a letrozole-induced PCOS rat model. Oral administration of the combined aqueous extracts (250 mg/kg for 21) improved the estrous cycle, reduced the number of cystic follicles by regulating ovarian folliculogenesis and increased estradiol and estrogen receptor (ESR1) expression, thereby preventing uterine shrinkage and restoring reproductive function. In addition to this, there was a decline in serum glucose and triglyceride levels, suggesting beneficial metabolic effects. Together, these findings indicate that Shatavari may influence hormonal and metabolic pathways relevant to PCOS pathophysiology, although further mechanistic and clinical validation is needed (8).

Based on this, preliminary preclinical evidence and its traditional use as a reproductive tonic suggest that Shatavari may enhance follicular development, improve oocyte quality, regulate hormonal secretion, and reduce oxidative stress via antioxidant activity (9). Therefore, this present pilot study aimed to evaluate the safety and efficacy of standardized Shatavari root extract in women with PCOS. The primary objective was to measure the changes in ovarian volume and follicle count, and endometrial thickness after 12 weeks of administration of Shatavari root extract supplementation. Secondary objectives were to evaluate the changes in laboratory parameters stress levels, metabolic and hormonal profiles.

## Methods

### Study design

This was a 12-week, prospective, randomized, double-blind, two-arm, parallel, placebo-controlled pilot trial. The clinical study protocol was approved by the Institutional Ethics Committee (IEC) of Dr. D. Y. Patil Medical College & Hospital, Navi Mumbai, Maharashtra, India (IEC Reference No: DYP/IECBH/2024/425). The study was registered with the Clinical Trials Registry of India (CTRI) with registration number CTRI/2024/10/074660 on October 3, 2024. The study followed the principles described in the Declaration of Helsinki (2013 revision) and was conducted in compliance with Good Clinical Practice (GCP) guidelines and the Consolidated Standards of Reporting Trials (CONSORT) declaration.

Written informed consent from all participants was obtained in their preferred languages (Hindi, Marathi, and English) before enrolment. Before obtaining consent, each participant was provided a comprehensive explanation of the study’s goal and the anticipated outcome.

### Study population

#### Inclusion criteria

Women aged 20–40 years presenting to the study site with clinical signs and symptoms suggestive of PCOS were screened for eligibility. Participants were included if they consented to undergo a transvaginal ultrasound for ovarian assessment, understood the study requirements, agreed to follow the procedure, and were willing to sign an informed consent and comply with all study protocols.

PCOS diagnosis was based on ovarian morphology assessed by transvaginal ultrasound, in accordance with the Rotterdam criteria and consistent with current European Society of Human Reproduction and Embryology (ESHRE) recommendations. Polycystic ovarian morphology was defined as the presence of an ovarian volume >10 cm³ and/or micro-polycystic ovaries (≥12 follicles per ovary measuring 2–9 mm) in at least one ovary (6, 7). Additional inclusion criteria were having no hormonal treatment within the past six months, a luteinizing hormone (LH) to follicle-stimulating hormone (FSH) ratio > 2.5, and no history or current diagnosis of adrenal or other endocrine disorders.

#### Exclusion criteria

Women with known allergies to Shatavari were excluded. Additionally, women with congenital adrenal hyperplasia, androgen-secreting tumors, Cushing’s syndrome, a history of any hormonal therapy within the past six months, hypothyroidism, twins, or higher-order births, as well as those with significant endocrine, metabolic, hepatic, renal, cardiovascular, gastrointestinal, respiratory, hematological, or neurological illnesses, and current psychiatric disorders were not included in the study. Women who had used any investigational drug within three months before the study were also excluded.

### Sample size calculation

The sample size calculations were based on the primary outcome, i.e., change in ovarian volume (mL) from baseline to the conclusion with herbal treatment or placebo. The reported ovarian volume following 12 weeks of resveratrol (*Polygonum cuspidatum*) administration (1000 mg/day) was 14.88 mL (3.83) at baseline and 12.83 mL (3.50) after 12 weeks. Thus, the decrease (p = 0.094) in ovarian volume was -2.05 mL (3.67, 95% confidence interval (CI) -4.46 to 0.36). The authors reported ovarian volumes of 12.72 mL (3.63) at baseline and 13.51 mL (3.79) after 12 weeks of placebo treatment (mean change = 0.79, standard deviation SD = 3.71). The reduction in ovarian volume with Shatavari was expected to be equivalent to resveratrol therapy, as reported by Taheri APM et al. (2022) (10). As no prior interventional data were available for Shatavari in PCOS at the time of study design, effect size assumptions were extrapolated from a published randomized trial evaluating resveratrol (Polygonum cuspidatum) in women with PCOS, which assessed ovarian volume using comparable ultrasonographic methods. This approach was used to estimate a clinically meaningful difference for ovarian volume in the absence of Shatavari-specific data. A sample size of 30 in each group achieves 90.1% power to detect a difference of -2.1 (active versus placebo), with the alternative hypothesis that the mean of group 2 (placebo) was 0.79, using known standard deviations of 3.67 and 3.71 and a two-independent-sample t-test. The significance level (alpha) was 0.050 for a one-sided two-sample t-test. Assuming a dropout rate of about 15% at the end of 12 weeks, 70 participants were planned to be enrolled in the study (35 in each group).

### Randomization and blinding

Randomization was carried out using an automated random number generation system (Rando version 1.2 R), pre-specified for the study. To maintain blinding, the Shatavari root extract and placebo capsules were identical in appearance, shape, color, and packaging. The randomization codes were securely store in separate envelopes, and were only accessed by the investigator after assigning a study number to each participant. The investigator and personnel were responsible for data collection and statistical analysis, and remained blinded to the treatment allocation throughout the study.

### Study interventions

Participants were randomized in a 1:1 ratio as per the randomization schedule to receive either a Shatavari capsule (a light brown capsule containing 300 mg Shatavari root extract) (Ixoreal Biomed Inc., Los Angeles, USA) or an identical placebo capsule (a light brown capsule containing 300 mg starch. Participants were instructed to take one capsule after a meal, once daily with water, for 12 weeks.

### Investigational products

#### Product details

The Shatavari root extract utilized in this study was derived from cultivated roots grown in sandy, dry soil with a pH range of 7.0–8.0, conditions optimized to enhance the concentration of bioactive constituents, such as total Shatavarins. This extract was prepared in alignment with green chemistry principles devoid of harsh solvents. The final product had an herb to extract ratio of 13:1 and was standardized to contain a minimum > 10% total Shatavarins, as determined by high-performance liquid chromatography (HPLC). The resulting extract was a yellowish-brown powder encapsulated for oral administration.

Participants in the placebo group received inert starch, matched to the investigational product in size, shape, color, and taste as the Shatavari capsule.

### Study assessments

Demographic characteristics and medical history were recorded at the baseline. Vital signs were monitored at every visit. Transvaginal ultrasound scans were performed at baseline, week 8, and week 12. BMI was measured at baseline, week 4, week 8, and week 12. PSS assessments were conducted at baseline, week 4, week 8, and week 12. Laboratory tests were carried out at baseline and week 12.

#### Primary outcome measure

##### Ovarian and endometrial outcomes

An internal transvaginal ultrasound scan is a crucial diagnostic tool for PCOS. It provides detailed images of the ovaries and helps identify key features of PCOS, such as multiple small cysts or follicles and enlarged ovarian volume. The scan also measures endometrial thickness, which is important for managing PCOS. According to the revised Rotterdam criteria, a PCOS diagnosis requires at least two of the following: oligo/anovulation, clinical or biochemical signs of hyperandrogenism, and polycystic ovaries on ultrasound (11).

The intravaginal ultrasound scan was conducted using a transducer frequency of 7.5 MHz. After obtaining a written informed consent form, the patient, with an empty bladder, lies on her back with her feet in stirrups. A lubricated, sterile ultrasound probe is carefully inserted into the vagina, producing sound waves that provide images of the ovaries and uterus. The images were analyzed using a normal ultrasound procedure for follicle count, ovarian volume, and endometrial thickness.

##### Secondary outcomes measure

BMI was measured at baseline, week 4, week 8, and week 12.

#### Laboratory assessments

Blood samples were collected at baseline and week 12 to evaluate the changes in HbA1c, lipid profile (total cholesterol, high-density lipoprotein cholesterol [HDL], low-density lipoprotein cholesterol [LDL], and triglycerides), serum insulin by HOMA-IR, serum hormones (follicle stimulating hormone (FSH), luteinizing Hormone (LH), estradiol, testosterone, and dehydroepiandrosterone sulfate [DHEA-S]), liver parameters (alanine transaminase [ALT], aspartate transaminase [AST], alkaline phosphatase [ALP], and bilirubin), renal parameters (creatinine and blood urea nitrogen), and thyroid parameters (triiodothyronine [T3], Thyroxine [T4], and thyroid-stimulating hormone [TSH]). Chemiluminescent immunoassay (CMIA) was used to conduct blood laboratory tests. These assessments were conducted to detect any adverse impacts on vital organs and for the safety of participants throughout the study.

#### Change in perceived stress scale

The Perceived Stress Scale (PSS-10) is the most widely adopted psychological tool for gauging stress perception (12). It evaluates the extent to which situations in one’s life are perceived as stressful. Specifically, it assesses how unpredictable, uncontrollable, and overwhelming respondents feel. The scale comprises several questions about feelings and thoughts experienced in the past month, which ask how frequently certain emotions were felt. Responses are scored from 0 (Never), 1 (Almost Never), 2 (Sometimes), 3 (Often), to 4 (Very Often). For questions 4, 5, 7, and 8, the scores are reversed (0 = 4, 1 = 3, 2 = 2, 3 = 1, 4 = 0). The overall score reflects the level of stress: 0–13 indicates low stress, 14–26 indicates moderate stress, and 27–40 indicates high stress.

#### Adverse events

As a safety assessment, the study recorded the Treatment-Emergent Adverse Events (TEAEs) and Treatment-Emergent Serious Adverse Events (TESAEs), either noticed by the physician or reported by the participants.

### Statistical methods and data analysis

Data from all participants who received at least one dose of study supplement (ITT: intent-to-treat) were used to assess safety and tolerability outcomes, whereas efficacy assessments were analyzed on the per-protocol (PP) dataset. All essential statistical computations were performed with the Statistical Package for Social Sciences (SPSS) software (version 21, IBM Corporation, USA). The analytical results for ranking data and scores were presented as mean ± SD. To verify that the best statistical techniques were followed, the study used 95% confidence intervals (CI). All analyses used two-sided testing, and a p-value less than 0.05 was considered statistically significant. Between-group comparisons were analyzed using an independent sample t-test. For categorical variables, we provided the number and percentage of individuals in each category of the parameters (including a category for missing data if needed) and calculated p-values using the chi-square test to compare the groups (SHT vs. PL). Multiple secondary outcomes were assessed without formal adjustment for multiplicity; therefore, the possibility of type I error should be considered when interpreting statistically significant findings for these outcomes.

## Results

**Figure 1:**
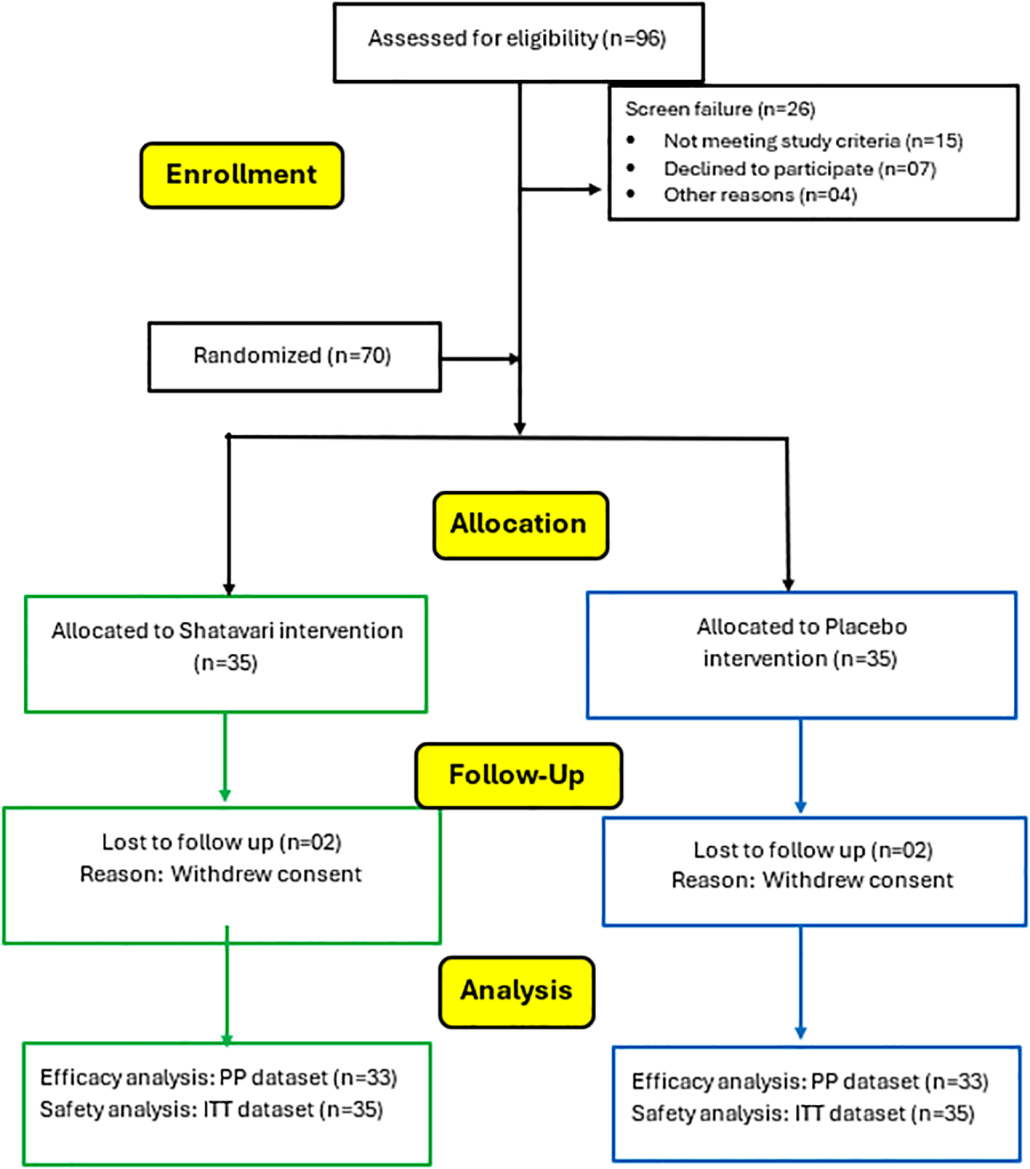
CONSORT chart.

A total of 96 women were assessed for eligibility, of whom 15 did not meet the inclusion criteria, 7 declined to participate, and 4 were excluded for other reasons. Hence, seventy women were randomized to receive SHT (n = 35) or placebo (PL; n = 35). During the study, four participants withdrew consent and were lost to follow-up, including two in the SHT group and two in the PL group. Therefore, the per-protocol (PP) population for efficacy analyses comprised 66 participants (SHT, n = 33; PL, n = 33). All 70 randomized participants were included in the intention-to-treat (ITT) population for safety analyses. **Figure 1** depicts the consort chart.

### Demographic and baseline data

**Table 1** presents a comparison of demographic and clinical parameters between the two groups. Both groups have similar demographics, with no significant differences in age. Psychological stress levels, reproductive health indicators, hormonal profiles, glycemic and metabolic profiles, lipid profiles, liver function tests, serum protein levels, renal function tests, and thyroid function tests show no significant differences between the groups.

**Table 1:** Demography and baseline profile in ITT dataset (n=70).

Baseline parameters	Shatavari (n=35)	Placebo (n=35)	p*
	Mean (SD)	Mean (SD)	
Demography			
• Age	27.97 (4.29)	27.66 (5.36)	0.787
• BMI (Kg/m2)	24.60 (4.10)	24.48 (4.42)	0.905
Psychological stress			
• PSS Total Score	34.89 (1.68)	34.91 (2.08)	0.950
Reproductive health			
• Ovarian Volume (ml)	17.14 (3.11)	17.09 (3.30)	0.941
• Follicle Count	13.34 (1.33)	13.37 (1.06)	0.921
• Endometrial Thickness (mm)	5.14 (2.05)	5.14 (1.83)	1.000
Hormonal profile			
• Estradiol (pg/ml)	219.17 (23.33)	219.89 (30.10)	0.912
• FSH (IU/L)	24.94 (8.09)	24.66 (8.62)	0.887
• LH (IU/L)	64.89 (12.75)	67.37 (12.66)	0.416
• Total Testosterone (ng/dL)	106.34 (11.93)	109.43 (12.83)	0.301
• Progesterone (ng/ml)	47.60 (7.47)	46.69 (7.38)	0.608
• DHEA-S (µg/dL)	29.32 (10.60)	30.07 (8.01)	0.740
Glycemic and metabolic profile			
• HbA1c (%)	6.13 (0.49)	6.14 (0.42)	0.933
• Plasma Glucose (mg/dL)	91.31 (15.42)	91.40 (11.20)	0.979
• Insulin (μU/mL)	61.63 (22.14)	61.91 (16.99)	0.952
Lipid profile			
• LDL-C (mg/dL)	120.37 (27.90)	120.20 (29.73)	0.980
• HDL-C (mg/dL)	50.37 (7.30)	48.40 (8.93)	0.315
• Total Cholesterol (mg/dL)	178.49 (27.90)	178.31 (29.81)	0.980
• Triglycerides (mg/dL)	113.23 (4.92)	113.74 (23.98)	0.902
Liver function tests			
• Total Bilirubin (mg/dL)	2.76 (0.77)	2.76 (0.77)	0.994
• Direct Bilirubin (mg/dL)	1.40 (0.40)	1.43 (0.40)	0.767
• Indirect Bilirubin (mg/dL)	1.36 (0.41)	1.33 (0.44)	0.768
• Alkaline Phosphatase (IU/L)	137.09 (3.49)	138.54 (7.39)	0.298
• AST (IU/L)	40.77 (15.58)	40.82 (11.08)	0.988
• ALT (IU/L)	44.21 (10.90)	44.15 (15.27)	0.984
Serum protein levels			
• Total Protein (g/dL)	7.71 (2.21)	7.02 (0.82)	0.086
• Albumin (g/dL)	3.88 (0.74)	3.87 (0.46)	0.926
• Globulin (g/dL)	2.91 (0.68)	2.87 (1.08)	0.860
Renal function tests			
• Creatinine (mg/dL)	0.75 (0.13)	0.72 (0.19)	0.427
• BUN (mg/dL)	67.00 (24.92)	61.49 (19.08)	0.302
Thyroid function tests			
• TSH (mIU/L)	8.78 (3.27)	8.72 (2.69)	0.932
• T3 (ng/dL)	3.71 (1.49)	3.86 (1.54)	0.694
• T4 (ng/dL)	3.94 (1.37)	3.97 (1.48)	0.934

*p‐value was obtained using an independent two‐sample t‐test for differences between two means (two‐tailed).

BMI, Body mass index; ITT, intent-to-treat; SHT, Shatavari; PSS, Perceived Stress Scale; SD, Standard deviation; FSH, Follicle-stimulating hormone; LH, Luteinizing hormone; PP, per-protocol; SD, Standard deviation; AST, Aspartate aminotransferase; ALT, Alanine transaminase; TSH, Thyroid-stimulating hormone; T3, Triiodothyronine Serum; T4, Thyroxine Serum.

### Ovarian and endometrial outcomes

**Figure 2:**
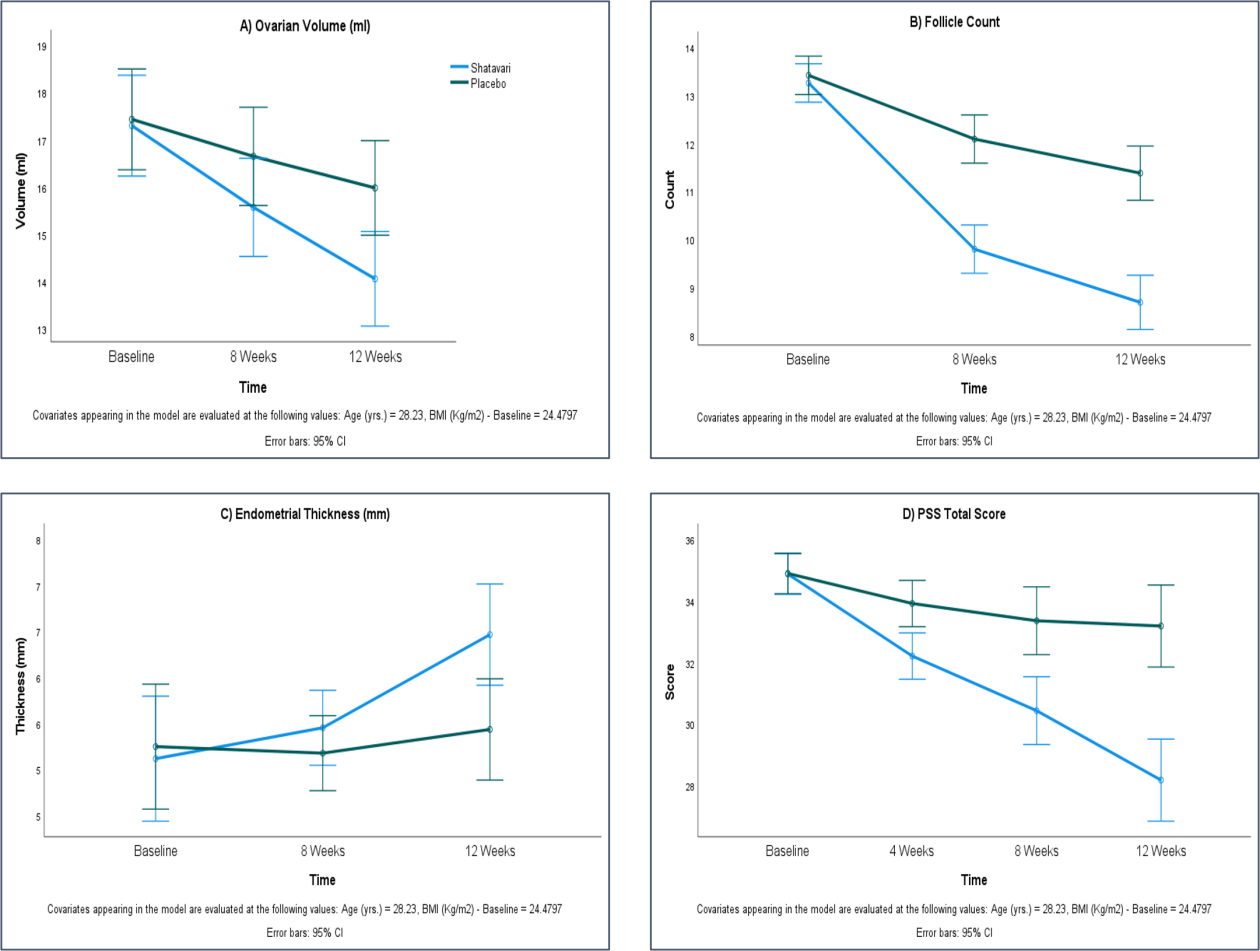
Comparative effects of therapy on ovarian ultrasound values and PSS total score in the PP dataset (n=66).

Over the 12-week intervention, ovarian volume showed a reduction in the SHT group compared with PL (−3.06 ± 6.83 mL vs −1.09 ± 7.06 mL), but the difference was not statistically significant (p = 0.254). Both primary outcomes, follicle count and endometrial thickness, showed significant improvements in the SHT group at week 12. Follicle count decreased by −3.79 ± 1.05 versus −2.39 ± 0.87 in PL (p < 0.0001), and endometrial thickness increased by 1.33 ± 2.23 mm versus 0.20 ± 1.84 mm in PL (p = 0.028). These results indicate that SHT supplementation was associated with meaningful improvements in reproductive parameters, despite ovarian volume not reaching statistical significance (**Table 2**, **Figure 2**).

**Table 2:** BMI, PSS score, and ovarian ultrasound findings in PP dataset (n=66).

Parameters	Shatavari (n=33)	Placebo (n=33)	Difference	Unpaired t-test		Cohen’s d
	*Mean (SD.)*	*Mean (SD.)*	*Mean (95% C.I.)*	*t*	*p	*(95% C.I.)*
Anthropometry						
BMI (Kg/m^2^)						
• Baseline	24.43 (4.03)	24.53 (4.54)	-0.11 (-2.22 to 2.01)	-0.101	0.920	-0.025 (-0.507 to 0.458)
• Change at 4 Weeks	-0.22 (3.43)	-0.11 (5.19)	-0.11 (-2.28 to 2.05)	-0.105	0.916	-0.026 (-0.508 to 0.457)
• Change at 8 Weeks	-0.52 (2.66)	-0.13 (4.52)	-0.39 (-2.21 to 1.43)	-0.426	0.671	-0.105 (-0.587 to 0.378)
• Change at 12 Weeks	-1.07 (4.30)	-0.29 (3.68)	-0.78 (-2.75 to 1.19)	-0.789	0.433	-0.194 (-0.677 to 0.290)
Psychological stress						
PSS total score						
• Baseline	34.85 (1.72)	34.91 (2.11)	-0.06 (-1.01 to 0.89)	-0.128	0.899	-0.031 (-0.514 to 0.451)
• Change at 4 Weeks	-2.64 (2.61)	-1.00 (3.24)	-1.64 (-3.08 to -0.19)	-2.260	0.027	-0.556 (-1.046 to -0.062)
• Change at 8 Weeks	-4.42 (2.95)	-1.55 (3.97)	-2.88 (-4.60 to -1.16)	-3.345	0.001	-0.823 (-1.324 to -0.317)
• Change at 12 Weeks	-6.64 (3.99)	-1.76 (5.21)	-4.88 (-7.16 to -2.60)	-4.271	<0.0001	1.052 (-1.564 to -0.532)
Reproductive health						
Ovarian volume (ml)						
• Baseline	17.27 (3.15)	17.45 (3.02)	-0.18 (-1.70 to 1.33)	-0.239	0.812	-0.059 (-0.541 to 0.424)
• Change at 8 Weeks	-1.67 (7.37)	-0.21 (10.56)	-1.45 (-5.93 to 3.02)	-0.649	0.519	-0.160 (-0.642 to 0.324)
• Change at 12 Weeks	-3.06 (6.83)	-1.09 (7.06)	-1.97 (-5.39 to 1.45)	-1.152	0.254	-0.284 (-0.767 to 0.203)
Follicle count						
• Baseline	13.24 (1.28)	13.42 (1.06)	-0.18 (-0.76 to 0.40)	-0.629	0.531	-0.155 (-0.638 to 0.329)
• Change at 8 Weeks	-2.39 (2.91)	-1.80 (1.02)	-0.59 (-1.67 to 0.48)	-1.099	0.276	-0.271 (-0.754 to 0.215)
• Change at 12 Weeks	-3.79 (1.05)	-2.39 (0.87)	-1.39 (-1.87 to -0.92)	-5.853	<0.0001	-1.441 (-1.979 to -0.893)
Endometrial thickness (mm)						
• Baseline	5.12 (2.01)	5.24 (1.84)	-0.12 (-1.07 to 0.83)	-0.256	0.799	-0.063 (-0.545 to 0.420)
• Change at 8 Weeks	0.33 (2.23)	-0.07 (1.64)	0.40 (-0.56 to 1.36)	0.835	0.407	0.206 (-0.279 to 0.689)
• Change at 12 Weeks	1.33 (2.23)	0.20 (1.84)	1.13 (0.13 to 2.14)	2.253	0.028	0.555 (0.061 to 1.044)

Within-group comparison: Ovarian Volume and Follicle Count: Both Shatavari and Placebo demonstrated significant improvements at 8 and 12 weeks (p<0.0001). Endometrial Thickness: Shatavari showed a significant increase at 12 weeks (p = 0.002), while Placebo exhibited no significant change (p = 0.536). PSS total score: Shatavari showed significant improvements at all time points (p <0.0001), whereas Placebo had only minimal effects (p = 0.032 at week 8, p = 0.061 at week 12). BMI: No significant changes were observed in BMI for either group (p > 0.05 at all time points). *p‐value was obtained using an independent two‐sample t‐test for differences between two means (two‐tailed). BMI, Body mass index; SHT, Shatavari; PSS, Perceived Stress Scale; SD, Standard deviation; mL, Milliliters; mm.

### BMI and PSS score

Across the 12-week intervention, no significant differences in BMI changes were observed between the groups at any time point, with both groups showing minimal, non-significant reductions (**Table 2**). The psychological stress demonstrated a consistent and significant improvement with SHT supplementation. By week 4, the SHT group showed a greater reduction in PSS scores compared to placebo (p = 0.027). This effect was maintained and further strengthened at week 8 (−4.42 ± 2.95 vs −1.55 ± 3.97; p = 0.001) and week 12 (−6.64 ± 3.99 vs −1.76 ± 5.21; p < 0.0001) (**Figure 2**).

### Serum hormones

**Table 3** shows no statistically significant differences in the levels of estradiol, FSH, LH, testosterone, progesterone, or DHEA-S between the SHT and PL groups at baseline or after 12 weeks.

**Table 3:** Serum hormones in PP dataset (n=66).

Parameters	Shatavari (n=33)	Placebo (n=33)	Difference	Unpaired t-test		Cohen’s d
	*Mean (SD)*	*Mean (SD)*	*Mean (95% C.I.)*	*t*	*p*	*(95% C.I.)*
Serum hormone						
Estradiol (pg/mL)						
• Baseline	218.82 (23.99)	223.21 (24.10)	-4.39 (-16.22 to 7.43)	-0.742	0.461	-0.183 (-0.666 to 0.302)
• 12 Weeks	218.12 (23.83)	219.33 (24.14)	-1.21 (-13.01 to 10.58)	-0.205	0.838	-0.051 (-0.533 to 0.432)
FSH (IU/L)						
• Baseline	25.30 (8.11)	24.55 (8.87)	0.76 (-3.42 to 4.94)	0.362	0.718	0.089 (-0.394 to 0.572)
• 12 Weeks	23.02 (7.60)	24.14 (8.80)	-1.12 (-5.16 to 2.93)	-0.551	0.583	-0.136 (-0.618 to 0.348)
LH (IU/L)						
• Baseline	63.82 (12.33)	67.03 (12.56)	-3.21 (-9.33 to 2.91)	-1.048	0.298	-0.258 (-0.742 to 0.227)
• 12 Weeks	62.95 (11.93)	66.70 (12.55)	-3.75 (-9.77 to 2.27)	-1.245	0.218	-0.306 (-0.791 to 0.180)
Total testosterone (ng/dL)						
• Baseline	105.76 (12.04)	109.48 (13.05)	-3.73 (-9.90 to 2.45)	-1.206	0.232	-0.297 (-0.781 to 0.190)
• 12 Weeks	105.57 (12.15)	108.86 (12.94)	-3.28 (-9.45 to 2.89)	-1.063	0.292	-0.262 (-0.745 to 0.224)
Progesterone (ng/mL)						
• Baseline	47.73 (7.30)	46.55 (7.57)	1.18 (-2.48 to 4.84)	0.645	0.521	0.159 (-0.325 to 0.642)
• 12 Weeks	46.34 (7.09)	46.08 (7.50)	0.25 (-3.34 to 3.84)	0.141	0.888	0.035 (-0.448 to 0.517)
DHEA-S (µg/dL)						
• Baseline	28.47 (9.99)	30.39 (7.98)	-1.92 (-6.37 to 2.52)	-0.865	0.390	-0.213 (-0.696 to 0.272)
• 12 Weeks	28.45 (9.98)	30.00 (7.91)	-1.55 (-5.98 to 2.88)	-0.699	0.487	-0.172 (-0.655 to 0.312)

*p‐value was obtained using an independent two‐sample t‐test for differences between two means (two‐tailed).

SHT, Shatavari; SD, Standard deviation; FSH, Follicle-stimulating hormone; LH, Luteinizing hormone; PP, per-protocol; pg/mL, Picograms per milliliter; IU/mL, International Units per milliliter; IU/L, International Units per litter; ng/dL, Nanograms per deciliter; ng/mL, Nanograms per milliliter; DHEA-S, Dehydroepiandrosterone sulfate; µg/dL, Micrograms per deciliter.

### Laboratory parameters

**Table 4** shows there were no statistically significant differences in the glycaemia and metabolic profiles, lipid profiles, liver function tests, protein levels, renal function tests, or thyroid function tests between the Shatavari and placebo groups at baseline or 12 weeks.

**Table 4:** Laboratory parameters in PP dataset (n=66).

Parameters	Shatavari (n=33)	Placebo (n=33)	Difference	Unpaired t test		Cohen’s d
	*Mean (SD)*	*Mean (SD)*	*Mean (95% C.I.)*	*t*	*p*	*Mean (95% C.I.)*
Glycemic and metabolic profile						
HbA1c (%)						
• Baseline	6.11 (0.49)	6.11 (0.41)	0.00 (-0.23 to 0.22)	-0.022	0.983	-0.005 (-0.488 to 0.477)
• 12 Weeks	6.08 (0.54)	6.12 (0.56)	-0.03 (-0.30 to 0.24)	-0.246	0.806	-0.061 (-0.543 to 0.422)
Plasma glucose (μU/mL)						
• Baseline	90.61 (15.09)	91.64 (11.30)	-1.03 (-7.59 to 5.52)	-0.314	0.755	-0.077 (-0.560 to 0.406)
• 12 Weeks	89.61 (15.09)	90.03 (10.34)	-0.42 (-6.78 to 5.94)	-0.133	0.894	-0.033 (-0.515 to 0.450)
Insulin (μU/mL)						
• Baseline	62.70 (22.33)	62.45 (16.89)	0.24 (-9.49 to 9.98)	0.050	0.960	0.012 (-0.470 to 0.495)
• 12 Weeks	61.30 (22.31)	61.64 (16.90)	-0.33 (-10.07 to 9.40)	-0.068	0.946	-0.017 (-0.499 to 0.466)
Lipid profile						
LDL (mg/dL)						
• Baseline	120.55 (28.58)	119.09 (30.28)	1.46 (-13.02 to 15.94)	0.201	0.841	0.050 (-0.433 to 0.532)
• 12 Weeks	119.03 (28.63)	118.97 (31.11)	0.06 (-14.64 to 14.77)	0.009	0.993	0.002 (-0.480 to 0.485)
HDL (mg/dL)						
• Baseline	50.61 (7.45)	48.67 (9.01)	1.94 (-2.13 to 6.00)	0.953	0.344	0.235 (-0.251 to 0.718)
• 12 Weeks	52.31 (5.40)	50.19 (8.74)	2.12 (-1.60 to 5.84)	1.138	0.260	0.289 (-0.213 to 0.789)
Cholesterol (mg/dL)						
• Baseline	178.55 (28.58)	177.09 (30.28)	1.46 (-13.02 to 15.94)	0.201	0.841	0.050 (-0.433 to 0.532)
• 12 Weeks	176.49 (28.55)	176.99 (30.28)	-0.50 (-14.98 to 13.97)	-0.069	0.945	-0.017 (-0.500 to 0.465)
Triglycerides (mg/dL)						
• Baseline	113.09 (5.04)	113.82 (24.69)	-0.72 (-9.49 to 8.04)	-0.165	0.869	-0.041 (-0.523 to 0.442)
• 12 Weeks	111.15 (22.71)	112.03 (24.94)	-0.88 (-12.60 to 10.85)	-0.149	0.882	-0.037 (-0.519 to 0.446)
Liver function tests						
Total bilirubin (mg/dL)						
• Baseline	2.71 (0.76)	2.80 (0.77)	-0.09 (-0.47 to 0.29)	-0.475	0.637	-0.117 (-0.599 to 0.367)
• 12 Weeks	2.68 (0.82)	2.73 (0.95)	-0.05 (-0.49 to 0.38)	-0.251	0.802	-0.062 (-0.544 to 0.421)
Direct bilirubin (mg/dL)						
• Baseline	1.38 (0.40)	1.44 (0.41)	-0.06 (-0.26 to 0.14)	-0.611	0.543	-0.150 (-0.633 to 0.333)
• 12 Weeks	1.38 (0.40)	1.44 (0.41)	-0.06 (-0.26 to 0.14)	-0.611	0.543	-0.150 (-0.633 to 0.333)
Indirect bilirubin (mg/dL)						
• Baseline	1.33 (0.41)	1.36 (0.42)	-0.03 (-0.23 to 0.17)	-0.283	0.778	-0.070 (-0.552 to 0.413)
• 12 Weeks	1.30 (0.51)	1.29 (0.67)	0.01 (- -0.29 to 0.30)	0.039	0.969	0.010 (-0.473 to 0.492)
Alkaline phosphatase (IU/L)						
• Baseline	136.88 (3.20)	138.73 (7.57)	-1.85 (-4.70 to 1.01)	-1.290	0.202	-0.318 (-0.802 to 0.169)
• 12 Weeks	133.58 (13.94)	136.92 (7.77)	-3.34 (-8.89 to 2.21)	-1.202	0.234	-0.296 (-0.780 to 0.190)
AST (IU/L)						
• Baseline	40.33 (15.95)	40.58 (11.33)	-0.25 (-7.05 to 6.56)	-0.073	0.942	-0.018 (-0.500 to 0.465)
• 12 Weeks	39.33 (15.95)	40.55 (10.79)	-1.22 (-7.92 to 5.48)	-0.363	0.718	-0.089 (-0.572 to 0.394)
ALT (IU/L)						
• Baseline	44.21 (11.23)	44.15 (15.74)	0.05 (-6.67 to 6.78)	0.016	0.987	0.004 (-0.479 to 0.486)
• 12 Weeks	42.18 (11.93)	42.20 (14.39)	-0.02 (-6.52 to 6.48)	-0.007	0.995	-0.002 (-0.484 to 0.481)
Protein levels						
Total protein (g/dL)						
• Baseline	7.68 (2.28)	7.04 (0.83)	0.64 (-0.20 to 1.49)	1.528	0.131	0.376 (-0.112 to 0.862)
• 12 Weeks	7.53 (2.36)	6.98 (0.86)	0.55 (-0.32 to 1.43)	1.269	0.209	0.312 (-0.174 to 0.797)
Albumin (g/dL)						
• Baseline	3.85 (0.75)	3.87 (0.47)	-0.02 (-0.32 to 0.29)	-0.102	0.919	-0.025 (-0.508 to 0.457)
• 12 Weeks	3.57 (1.05)	3.81 (1.24)	-0.24 (-0.81 to 0.32)	-0.864	0.391	-0.213 (-0.696 to 0.272)
Globulin (g/dL)						
• Baseline	2.88 (0.70)	2.92 (1.10)	-0.04 (-0.49 to 0.42)	-0.155	0.877	-0.038 (-0.521 to 0.444)
• 12 Weeks	2.66 (0.68)	2.81 (0.71)	-0.15 (-0.49 to 0.19)	-0.868	0.388	-0.214 (-0.697 to 0.271)
Renal function tests						
Creatinine (mg/dL)						
• Baseline	0.74 (0.13)	0.74 (0.17)	0.00 (-0.07 to 0.08)	0.081	0.936	0.020 (-0.463 to 0.502)
• 12 Weeks	0.63 (0.20)	0.67 (0.22)	-0.04 (-0.14 to 0.06)	-0.797	0.428	-0.196 (-0.679 to 0.288)
BUN (mg/dL)						
• Baseline	66.52 (24.83)	60.67 (18.83)	5.85 (-4.99 to 16.69)	1.078	0.285	0.265 (-0.220 to 0.749)
• 12 Weeks	64.27 (24.87)	60.65 (18.83)	3.63 (-7.22 to 14.47)	0.668	0.507	0.164 (-0.320 to 0.647)
Thyroid function tests						
TSH (mIU/L)						
• Baseline	8.72 (3.26)	8.61 (2.66)	0.11 (-1.36 to 1.57)	0.144	0.886	0.035 (-0.447 to 0.518)
• 12 Weeks	8.38 (3.13)	8.53 (2.63)	-0.15 (-1.57 to 1.27)	-0.208	0.836	-0.051 (-0.534 to 0.432)
T3 (ng/dL)						
• Baseline	3.67 (1.47)	3.82 (1.57)	-0.15 (-0.90 to 0.60)	-0.404	0.687	-0.100 (-0.582 to 0.384)
• 12 Weeks	3.52 (1.42)	3.78 (1.55)	-0.26 (-0.99 to 0.48)	-0.697	0.489	-0.172 (-0.654 to 0.313)
T4 (ng/dL)						
• Baseline	3.97 (1.40)	3.91 (1.49)	0.06 (-0.65 to 0.77)	0.170	0.865	0.042 (-0.441 to 0.524)
• 12 Weeks	3.85 (1.36)	3.95 (1.35)	-0.10 (-0.77 to 0.57)	-0.298	0.766	-0.073 (-0.556 to 0.410)

*p‐value was obtained using an independent two‐sample t‐test for differences between two means (two‐tailed).

SHT, Shatavari; SD, Standard deviation; PP, per-protocol; AST, Aspartate aminotransferase; ALT, Alanine transaminase; TSH, Thyroid-stimulating hormone; T3, Triiodothyronine Serum; T4, Thyroxine Serum; ALP, Alkaline Phosphatase.

### Adverse events

No serious adverse events were reported in either group. Mild to moderate adverse events occurred in 4 participants (11.4%) in the Shatavari (SHT) group and 3 participants (8.5%) in the placebo (PL) group. In the SHT group, reported events included nausea, headache, mood swings, and dyslipidemia, whereas participants in the PL group experienced headache, anxiety, and vomiting. Dyslipidemia was observed in a participant with a pre-existing lipid disorder and was managed with statin therapy; mood swings were reported in a participant with a prior history of affective symptoms and were treated with fluoxetine. All adverse events were transient or managed during the study period and did not lead to treatment discontinuation. Causality and severity were assessed by the study investigators, and all events were determined to be unrelated to the study intervention. Pharmacological treatments were initiated or continued according to standard clinical care and, where applicable, were continued beyond the study period at the discretion of the treating physician.

## Discussion

The present randomized, double-blind, placebo-controlled trial evaluated the effect of standardized Shatavari root extract on ovarian and endometrial parameters, hormonal profile, and psychological stress in women with PCOS. The key findings were a significant increase in endometrial thickness, a significant reduction in follicular count, and a reduction in perceived stress levels in the SHT group compared to PL. No significant changes were observed in estradiol, FSH, LH, testosterone, progesterone, or DHEA. However, the primary endpoint, change in ovarian volume, did not differ significantly between groups, indicating that the intervention did not alter overall ovarian size.

Preclinical studies provide a strong rationale for the present clinical findings. Vishnuvardhan et al. (2022) demonstrated that an aqueous extract of *Asparagus racemosus* alleviated metabolic disturbances in a Wistar rat model of PCOS by normalizing glucose, lipid, and liver enzyme levels, indicating a mitigating effect on PCOS-associated metabolic dysfunction (7). Similarly, Ghosh et al. (2025) reported that oral administration of combined aqueous extracts of *Asparagus racemosus* and *Vitex negundo* (250 mg/kg for 21 days) improved estrous cyclicity, reduced cystic follicle formation, and enhanced estradiol and estrogen receptor (ESR1) expression in a letrozole-induced PCOS rat model (8). These effects were accompanied by reductions in serum glucose and triglyceride levels, suggesting beneficial hormonal and metabolic modulation. These preclinical findings support the ability of Shatavari to regulate ovarian folliculogenesis, enhance uterine health, and modulate key hormonal and metabolic pathways relevant to PCOS pathophysiology.

While the neutral effect on ovarian volume indicates that primary structural changes were not achieved, the observed improvements in follicle count and endometrial thickness reflect meaningful reproductive outcomes. The results of the present study align with these experimental observations, where women receiving SHT demonstrated favorable reproductive outcomes, including a significant reduction in follicle count and an increase in endometrial thickness compared to the placebo group.

Ayurvedic literature similarly recognizes Shatavari as a key rejuvenative herb for women’s reproductive health. Ayurveda attributes PCOS to an imbalance of doshas (biological energies) and accumulation of toxins (ama), leading to cyst formation and hormonal dysregulation (13, 14). Traditional management employs a multifaceted approach encompassing dietary and lifestyle interventions, herbal formulations, detoxification (panchakarma), stress management, and rejuvenation therapies. Shatavari is known as a “feminine tonic,” traditionally used to stimulate folliculogenesis, regulate menstrual cycles, alleviate dysmenorrhea, and promote fertility (15).

Clinical evidence aligns with these traditional and preclinical insights. Dayani et al. (2010) reported that Shatavari, in combination with other Ayurvedic herbs, significantly reduced PCOS symptoms by decreasing excessive body hair, reducing the polycystic appearance of the ovaries, improving follicular maturity, and helping to maintain a regular menstrual cycle (16). In the current study at 12-week, the Shatavari group exhibited a significant increase in endometrial thickness (p = 0.028) and a significant decrease in follicular count (p<0.0001). It is important to note that these improvements occurred despite no significant changes in ovarian volume, highlighting that the benefits observed pertain to primary reproductive parameters rather than overall ovarian size. The increase in endometrial thickness may reflect enhanced estrogenic responsiveness at the uterine level, providing a more receptive environment. Although reductions in follicular count and increases in endometrial thickness were observed, the clinical implications of these ultrasound-based changes should be interpreted cautiously. In women with PCOS, reduced follicular count may indicate improved follicular dynamics and reduced follicular arrest, while increased endometrial thickness is commonly considered a surrogate marker of enhanced endometrial receptivity and menstrual regularity. However, ovulatory function, menstrual cyclicity, and fertility outcomes were not directly assessed in this study and require confirmation in future trials. The lack of significant shifts in serum reproductive hormones indicates that Shatavari’s effects may not primarily occur through gross systemic hormonal modulation, but rather through local ovarian and uterine effects or via modulation of receptor sensitivity and tissue responsiveness (6, 17). Hormonal outcomes should be interpreted with caution, as circulating reproductive hormones were assessed at single time points without standardization to menstrual cycle phase. Given the known cyclical variability of gonadotropins and sex steroids in women with PCOS, this methodological constraint may have limited the ability to detect treatment-related changes and should be considered when interpreting the null hormonal findings.

Women often face psychological, physical, and physiological stress. Psychological stress can affect ovarian physiology and oocyte quality, leading to female reproductive health disorders (18). The increase of reactive oxygen species (ROS) (19) and thereby oxidative stress can induce the onset of PCOS in some women (20). Hence, the PSS-10 was assessed as a secondary outcome to measure the perception of stress. At 12 weeks, the Shatavari group demonstrated significant reductions in psychological stress, as measured by the PSS total score. The results showed that Shatavari root extract considerably decreases psychological stress while increasing endometrial thickness over time when compared to the placebo group.

Four participants (11.4%) in the Shatavari group and three (8.5%) in the placebo group experienced mild to moderate adverse effects. Neither group reported any serious adverse events among their participants. Preclinical studies have indicated that Shatavari is safe at higher doses (7, 8).

The study’s randomized, double-blind, placebo-controlled design and evaluation of both reproductive and psychological outcomes strengthen the reliability of its findings. The observed improvements in endometrial thickness, follicular dynamics, and psychological stress may be related to multiple physiological actions of Shatavari root extract. Experimental evidence indicates that Shatavari may influence ovarian folliculogenesis and uterine tissue through phytoestrogen-like activity and modulation of estrogen receptor responsiveness, thereby enhancing endometrial receptivity without substantial changes in circulating hormone levels. Its antioxidant and anti-inflammatory properties may reduce oxidative stress and local ovarian dysfunction, which are implicated in PCOS pathophysiology. The reduction in follicular count may reflect improved follicular maturation and reduced follicular arrest rather than suppression of ovarian function. In addition, the adaptogenic properties of Shatavari may contribute to stress reduction through regulation of the hypothalamic–pituitary–adrenal axis, indirectly supporting reproductive function. The results demonstrate that Shatavari supplementation may be a valuable adaptogen in managing symptoms in women with PCOS.

This study was limited by its modest sample size and relatively short 12-week duration, which restricts assessment of long-term efficacy and safety Hormonal parameters were measured at single time points rather than across cycles, which limits interpretability and may have reduced sensitivity to detect subtle treatment effects on endocrine outcomes, and the homogenous study cohort may limit generalizability to broader, more diverse PCOS populations.

Future studies involving larger and more diverse cohorts with longer follow-up durations are required to confirm these findings, evaluate dose–response relationships, assess long-term safety, and examine clinically relevant outcomes, including ovulation, menstrual regularity, fertility, and metabolic parameters. In addition, mechanistic investigations focusing on local ovarian and endometrial signaling pathways may further elucidate the tissue-specific actions of Shatavari in PCOS.

## Conclusion

In this randomized, double-blind, placebo-controlled trial, standardized Shatavari root extract significantly improved follicular count and endometrial thickness in women with PCOS, while ovarian volume remained unchanged. A significant reduction in perceived psychological stress was also observed. No clinically meaningful changes were detected in reproductive hormones or safety parameters, and no serious adverse events occurred, indicating a safe and effective supplement over 12 weeks. These findings suggest potential reproductive and psychological benefits of Shatavari root extract in PCOS.

## Data Availability

The raw data supporting the conclusions of this article will be made available by the authors, without undue reservation.
